# Global directions of change in moral norms: a test of the moral argument theory

**DOI:** 10.1098/rsos.241589

**Published:** 2025-04-09

**Authors:** Pontus Strimling, Kimmo Eriksson, Irina Vartanova, Joel Krueger, Isabela Hazin

**Affiliations:** ^1^ Institute for Futures Studies, Stockholm, Sweden; ^2^ Department of Women’s and Children’s Health, Uppsala University, Uppsala, Sweden; ^3^ Institute for Analytical Sociology, Linköping University, Linkoping, Sweden; ^4^ Mälardalen University, Västerås, Sweden

**Keywords:** cultural evolution, global norm change, morality, individualizing arguments, moral argument theory

## Abstract

How and why are moral norms for various issues changing across the globe? Moral argument theory uniquely addresses these questions by positing that moral norm change is driven by ‘individualizing’ arguments: concerns about harm, fairness and liberty. We test this theory in a preregistered study of the arguments for both sides of 33 moral issues for which the global directions and rates of norm change were estimated in available longitudinal survey data from 94 societies. We also use available cross-national data to estimate the extent to which each society relies more on individualizing arguments than other kinds of arguments. In support of the theory, norms’ justifiability by individualizing arguments was found to predict their global change, and the effect of individualizing arguments on norm change is stronger in societies that rely more strongly on such arguments. These findings demonstrate a fundamental pattern in the contemporary cultural evolution of morality and highlights the key role played by individualizing arguments.

## 1. Introduction

Moral norms change over time. Consider norms about unmarried couples living together, a practice that was considered scandalous in Sweden when, mainly in Stockholm, it began to occur in the mid-1800s [1]. The practice spread to the point that it has now long been the norm for Swedish couples to live together for years before getting married (or never marrying at all). The relaxation of norms on the issue of unmarried cohabitation has also been documented in other societies, such as the United States [2]. A study of 27 different moral issues, for which longitudinal opinion data were available both in the United States and the United Kingdom, found that the entire ‘profile’ of norm changes over the last half-century was almost identical in the two countries; for example, the joint American-British profile of moral norm changes includes that acceptance of homosexuality has increased fast, that acceptance of sexual relations before marriage has increased relatively slower, and that acceptance of the death penalty has decreased [3]. This finding motivates two questions. What causes the similarity between the profiles of moral norm changes in the United States and the United Kingdom? And should we expect other countries to have similar profiles of norm change too?

These questions are addressed by the moral argument theory, which gives an account of the contemporary cultural evolution of moral norms [4]. It says that we can understand shifts in the moral norms surrounding an act by analysing which arguments speak in favour of accepting it and which arguments speak against it. The purpose of this paper is to test the ability of moral argument theory to predict global norm change.

Before going into the details of this theory, note that there may be any number of factors contributing to changes in the norm on a specific moral issue in a specific country. For example, the emergence of unmarried cohabitation in Stockholm in the 1800s has been attributed to increasing costs of getting married [1]. However, an important strength of the moral argument theory is its wide scope: arguments are expected to influence norms on every moral issue in every society. By ignoring other factors, the same theory makes predictions about the entire profile of changes in norms across different issues such as prostitution, soft drugs, lying, immigrant rights, protest marches and homosexual relations. For each issue, the theory makes a specific prediction about the direction and relative rate at which the norm, operationalized as the average opinion in society, changes over time in a specific society. It also predicts the relative rates of change in moral norms in different societies. In this paper, we examine the accuracy of its global predictions about profiles of norm changes on all moral issues for which change can be estimated in available data.

### 1.1. The moral argument theory

Moral argument theory describes a cultural evolutionary process in which moral norms change due to the moral arguments in favour of or against a specific norm that people expose each other to. Importantly, in this process the change is endogenously generated, that is, it is not driven by anything else in society changing (including the arguments themselves, which are assumed fixed).

The premise, adopted from moral foundations theory [5,6] is that all arguments that people use when discussing moral norms can be boiled down to just a few moral foundations. There is some variation in how researchers define the set of moral foundations; here we use six: Harm, Fairness, Liberty, Loyalty, Authority and Purity/Sanctity [3,7,8]. These foundations are grouped into two types: individualizing and binding [9]. Harm, Fairness and Liberty are individualizing foundations because they focus on universal concerns for individual outcomes; the other, binding, foundations address concerns about violations of social structures and ideals. Many justifications of moral judgements can be conceived as drawing on these foundations. For example, when people argue that a certain act is cruel and therefore bad or that an act is good because it cares for the weak or vulnerable, they appeal to the Harm foundation. Table 1 illustrates how a long list of arguments may be categorized into moral foundations.

**Table 1.** A set of moral arguments and their categorization into moral foundations of different types. Note. The arguments have been used in previous work on moral argument theory [10] and are mostly adapted from the Moral Foundations Questionnaire [7].

arguments	moral foundation	type
we should not cause emotional suffering	Harm	individualizing
we should care for the weak and vulnerable	"	"
we should not be cruel	"	"
we should not use violence	"	"
we should not kill	"	"
we should not physically harm others	"	"
we should not treat people differently	Fairness	"
we should not act unfairly	"	"
we should not deny someone his or her rights	"	"
everyone should be free to do as they want	Liberty	"
we should not restrict people’s freedom of choice	"	"
everyone should be free to decide what group norms or traditions they want to follow	"	"
we should be loyal	Loyalty	binding
we should not betray our group or community	"	"
we should show love for our country	"	"
we should show respect for authority	Authority	"
we should conform to the traditions of society	"	"
we should not create disruptions to the order of society	"	"
we should not violate standards of purity and decency	Purity/Sanctity	"
we should avoid doing anything disgusting	"	"
we should act in a way that God would approve of	"	"

Moral argument theory is based on a few plausible assumptions. The first one concerns which moral arguments resonate with which people. Based on extensive survey research using various versions of the Moral Foundations Questionnaire (MFQ) [3,7], individualizing arguments are assumed to resonate with most people. In contrast, binding arguments only resonate with certain people (‘moral conservatives’) and not with others (‘moral liberals’), representing the poles of a continuum.

The second assumption is that people sometimes are exposed to other people’s arguments and, occasionally, change their minds when exposed to an argument that resonates with them. Note that this is not a strong assumption. It is *not* assumed that every opinion people have is grounded in arguments, nor that arguments underlie all opinion change. As we describe below, it is sufficient that individuals sometimes change opinions based on arguments that resonate with them to generate norm change.

The third assumption is that arguments are not used arbitrarily; only certain arguments can credibly be used to justify a specific opinion on a moral issue. For example, arguments in favour of accepting gay marriage are typically based on fairness and liberty, whereas arguments against accepting gay marriage are typically based on purity/sanctity and authority [10]. On a given issue, the opinion that is best justified by individualizing arguments is said to have an *argument advantage*. For example, on the issue of gay marriage, acceptance has an argument advantage because fairness and liberty are individualizing arguments. An opinion’s argument advantage can be quantified; the more individualizing arguments favour one opinion over the opposite opinion, the bigger its argument advantage.

Based on these assumptions, the following social dynamics follows by which the opinion with the argument advantage spreads in the population. First, consider how opinions change among liberals. Because only individualizing arguments resonate with liberals, liberals are more likely to be swayed by arguments for the opinion with the argument advantage than by arguments for the opposite opinion. Over time, the average opinion among liberals will therefore change toward the argument-advantaged opinion. Now consider how opinions change among conservatives. On any moral issue, there are arguments on both sides, either individualizing or binding, and all kinds of arguments are assumed to resonate equally well with conservatives. Hence, conservatives are assumed equally likely to be swayed by arguments in either direction. Consequently, how their opinions move will depend on how frequently they are exposed to arguments for each opinion, which is determined by the current distribution of opinions in the population. The distribution changes when liberals are swayed to the argument-advantaged opinion, and therefore opinions will move in this direction also among conservatives.

### 1.2. A computational model

The logic of this social dynamics has been formalized in a computational model [4,11–13]. In the model, there is a population of agents of two moral types called liberal and conservative. Let *q* represent the proportion of liberal agents so that the proportion of conservative agents is 1 − *q*. Agents hold moral opinions. Their opinions may change over time in a process that proceeds in discrete time steps. At each time step, there is a small probability *d* that a given agent is exposed to an argument for another agent’s opinion. If this opinion is different from the agent’s current opinion, the probability of being swayed depends on the agent’s type, which opinion has the argument advantage, and the size *a* (0 < *a* ≤ 1) of that advantage. For liberal agents, a switch *to* the argument-advantaged opinion has a probability *s* but the probability of switching *from* the argument-advantaged opinion is only (1 − *a*)*s*. For conservative agents, the switching probability is the same (*s*) in both directions. At time *t*, let *l_t_* and *c_t_* be the proportions holding the argument-advantaged opinion among liberal and conservative agents, respectively. Assuming that agents meet at random, and the population is so large that stochastic effects can be ignored, the change from time *t* to time *t* + 1 in the popularity of the argument-advantaged opinion among liberal agents can be written as follows:


(1.1) $$\begin{array}{ll} l_{t+1}-l_{t} & =d[s(1-l_{t})(ql_{t}+(1-q)c_{t})-(1-a)sl_{t}(1-(ql_{t}+(1q)c+t))]\\ & =ds[a(1-l_{t})(ql_{t}+(1-q)c_{t})-(1-a)(1-q)(l_{t}-c_{t})]. \end{array}$$

Equation (1.1) describes the proportion of liberal agents that switch to the argument-advantaged opinion *minus* the proportion of liberal agents that switch to the opposite opinion. In the second line, this expression is rewritten so that the first term is positive and the size of the second term depends on how much the popularity of the argument-advantaged opinion differs between liberal and conservative agents $\left(l_{t}-c_{t}\right)$. It follows that as long as that difference is not too large, the popularity of the argument-advantaged opinion among liberal agents will continue to grow. The change from time *t* to time *t* + 1 in the popularity of the argument-advantaged opinion among conservative agents can similarly be expressed as follows:


(1.2) $$c_{t-1}-c_{t}=d[s(1-c_{t})(ql_{t}+(1-q)c_{t})-sc_{t}(1-(ql_{t}+(1-q)c_{t}))]=dsq(l_{t}-c_{t}).$$

**Figure 1.**
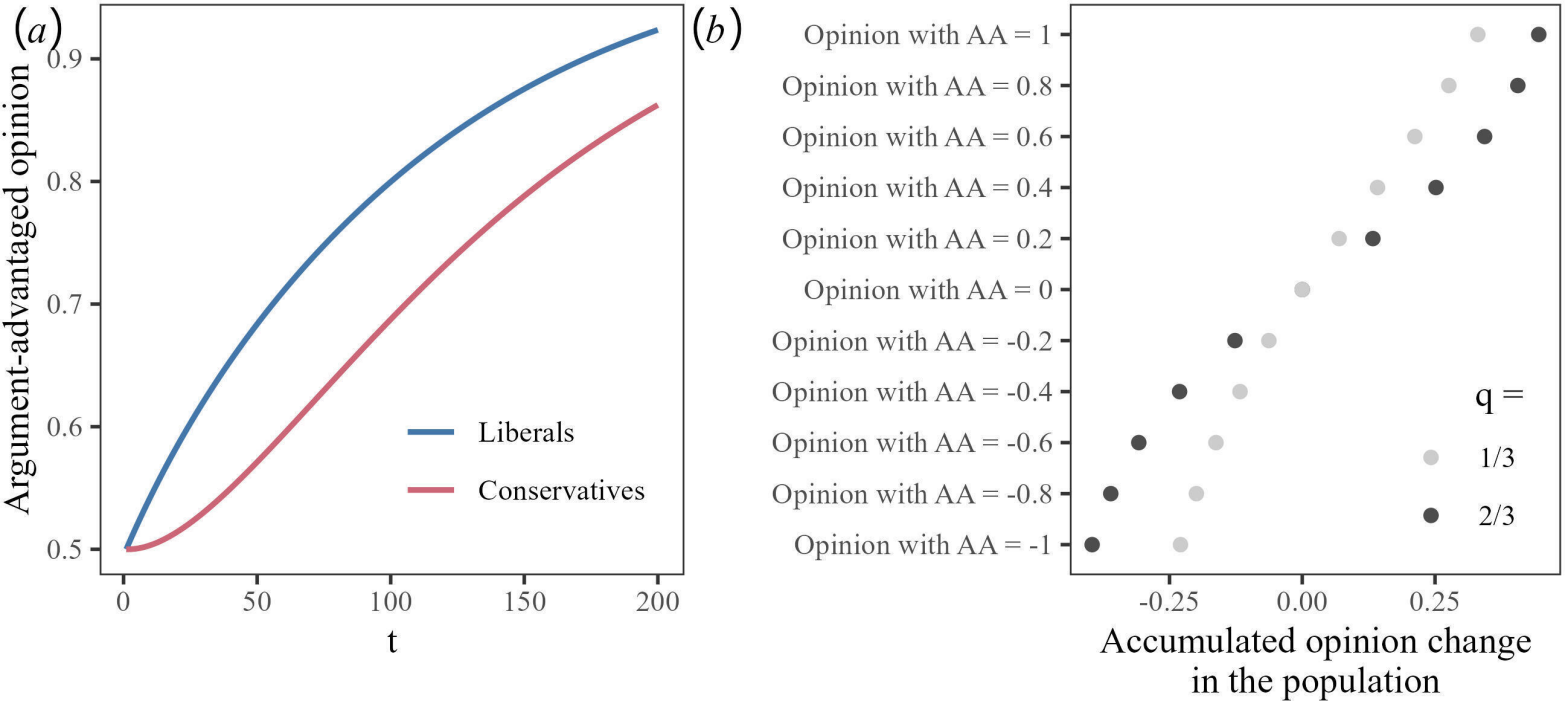
Model predictions of norm change over 200 time steps in the model when starting from a state in which the popularity of the argument-advantaged opinion is 50% among both liberal and conservative agents. (*a*) Predicted trajectories for liberals and conservatives. (*b*) The predicted profiles of norm change rates (accumulated over the first 100 time steps)—when the population is two-thirds liberals (black dots) or only one-third liberals (grey dots)—for opinions on 10 moral issues with different argument advantages. A negative argument advantage means that the opposite opinion has the argument advantage.

From equation (1.2), it follows that the popularity of the argument-advantaged opinion among conservative agents will continue to grow as long as it is below the corresponding popularity among liberal agents. The two equations together produce a dynamic in which the popularity of the argument-advantaged opinion (eventually) will gradually increase both among liberal and conservative agents (see figure 1*a*). The increase will be faster the larger the argument advantage is (model parameter *a*) and the more liberals there are in the population (model parameter *q*). Thus, for a selection of moral issues with argument advantages of different sizes, the model predicts a correspondingly shaped profile of norm change rates with a steeper slope the more liberals there are (see figure 1*b*).

### 1.3. Limitations of the theory

A limitation of the moral argument theory is that the notion of moral liberals who only rely on individualizing arguments is a simplification. There may be contexts when liberals think something is sacred [14]. When judging a target they dislike, liberals may find binding arguments relevant too [15]. Experimental studies of the immediate effects of exposure to various arguments on the opinions of liberals and conservatives have yielded mixed results [16–21]. However, a recent paper testing a broader set of issues supports the fundamental assumption that individualizing arguments are generally more persuasive than binding arguments, especially among moral liberals [22].

Another limitation is that arguments are not the only mechanism underlying moral judgements. According to social intuitionism, moral judgements are often shaped by intuition [23]. However, as mentioned earlier, such individual-level mechanisms may not matter much for population-level change in norms. The process of change outlined by moral argument theory will drive moral norms in the argument-advantaged direction as long as people are swayed by arguments at least occasionally. Unlike the social dynamics that arguments create, it is unclear how intuitions would drive population-level change in any direction. Hence, we do not expect the validity of the predictions from moral argument theory to be much affected by these additional factors—or any other factors that may influence individuals’ moral judgements but do not drive population-level change. This conclusion is supported by prior research finding the theory’s predictions about norm change in the United States and the United Kingdom to be highly accurate [11–13,24,25]. Thus, the key issue is whether moral argument theory is equally applicable in other societies around the world.

### 1.4. The applicability of the theory in different societies

To apply moral argument theory to societies across the globe, two questions need to be considered before we can make predictions. First, are the arguments for different opinions (and hence the argument advantage for one opinion over the opposite opinion) approximately the same in different societies and at different points in time? Prior research indicates an affirmative answer. A survey in four culturally diverse societies on different continents (the United States, United Kingdom, Brazil and Israel) found strong agreement within and between societies on which arguments justify which opinions on various moral issues [10]. In particular, this means that there is strong agreement on which opinion on an issue has the argument advantage (i.e. is best justified by individualizing arguments) [3]. Whether argument advantages are stable over time has not been tested directly. However, it is supported by the finding of stable opinion change trends on moral issues in the United States [25], as the theory predicts when argument advantages are stable.

Second, can every society’s population be approximated as a mix of people with whom only individualizing arguments resonate (moral liberals) and people with whom individualizing and binding arguments resonate equally (moral conservatives)? This question is addressed below through an examination of data from studies using the MFQ in societies around the world [26–28]. In every society, we find in these data that individualizing arguments are on average relied on more strongly than binding arguments, consistent with a mix of moral liberals and moral conservatives. We use the size of the difference in reliance on individualizing and binding arguments as a proxy for the proportion of liberals. This measure, henceforth called the *moral liberalness* of a society, varies across societies. It seems plausible that this variation is relatively stable over a few decades, which is the time span studied here.

In sum, there is evidence for the assumptions that the arguments for or against a specific moral norm are basically the same in different societies and at different points in time, and that there is a proportion of moral liberals in all societies, the size of which differs between societies. As discussed below, these assumptions allow us to derive predictions about global norm change and differences in change rates between societies.

### 1.5. Predictions

Based on the above discussion, we assume that the same set of argument advantage values applies in all societies and that these argument advantages drive norm change as outlined by moral argument theory in all societies. This yields several predictions about profiles of moral norm change in societies across the globe. The first prediction is that changes will tend to be similar in different societies.

**Prediction 1:** Different societies have similar profiles of moral norm change (i.e. across various moral issues, norm changes in different societies are correlated).

If this holds, it makes sense to conceive of a *global* profile of moral norm change that can be estimated by pooling data from different societies. Under the assumption that the argument advantages that drive the process are stable over time, this global profile is expected to be stable too.

**Prediction 2:** The global profile of moral norm change is consistent across different periods.

The key prediction is that there is a direct connection between the global profile of moral norm change and the argument advantage values that drive the process.

**Prediction 3:** The global profile of moral norm change correlates with the argument advantage values for the same set of moral norms.

In addition to these predictions about global norm change, the moral argument theory yields predictions of how norms and norm change vary across societies. The norm at any given time will reflect the sum of changes that have occurred up to that point. As shown in figure 1*b*, the theory says that changes (in the argument-advantaged direction) are faster in more morally liberal societies. Thus, assuming that the same process was ongoing already before the point when norms were first measured, the changes should have gone further in more morally liberal societies already at that point.

**Prediction 4:** At the first point of measurement, norms had already reached further in the argument-advantaged direction in more morally liberal societies.

These differences in moral change rates between societies are then assumed to continue throughout the period in which change can be observed.

**Prediction 5:** Observed norm change in the argument-advantaged direction is faster in more morally liberal societies.

### 1.6. Outline of study

The objective of this study is to test the above predictions from the moral argument theory. This involves collating data of three kinds. First, to estimate societies’ moral liberalness, we use available data from three cross-cultural studies of reliance on moral foundations.

**Figure 2.**
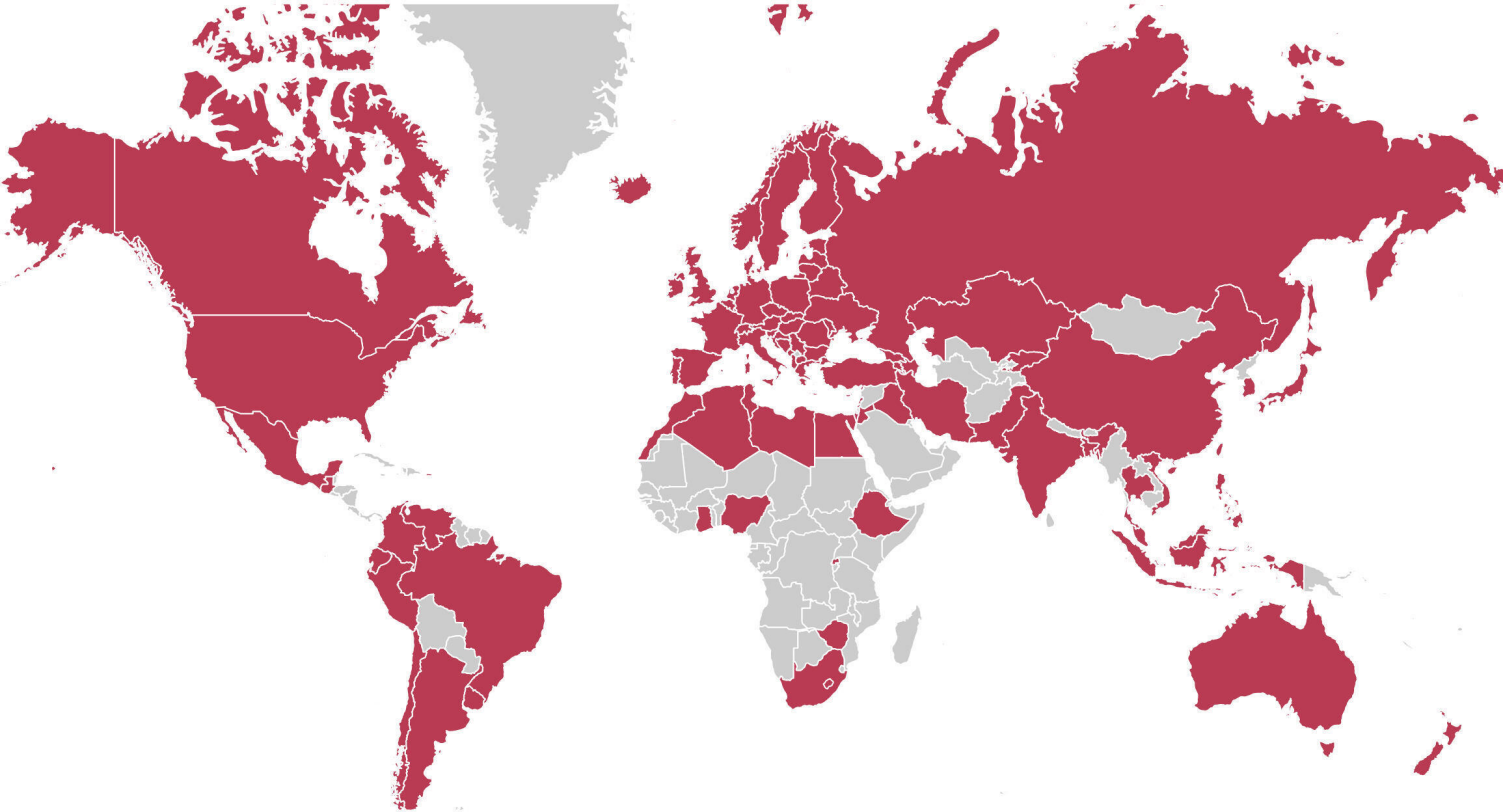
Global coverage of data on change of moral norms. For all coloured societies, longitudinal data on moral norms are available in the ISSP and/or the WVS/EVS.

Second, to estimate how norms for various moral issues have changed in societies across the globe requires cross-cultural, longitudinal opinion data on multiple moral issues. The largest available datasets that meet these criteria come from the International Social Survey Programme (ISSP) and the joint World Values Survey and European Values Survey (WVS/EVS). These surveys collect opinion data from nationally representative samples worldwide, with a new wave every few years since they started in the 1980s. By leveraging these datasets, we can track global shifts in moral norms for a wide range of behaviours such as extramarital sex, the death penalty and parental use of corporal punishment. Longitudinal data for such items are available in 94 societies, see figure 2, but every society does not have data on every item. In each society, the direction and speed of change in a given moral norm can be estimated from the longitudinal data. These estimates will inevitably be very noisy because of sampling errors at each point in time and, more importantly, because the number of time points is very small (sometimes just two points). However, noise is reduced by pooling norm change estimates in all societies with available data to obtain estimates of the global change in norms.

Third, to obtain measures of the argument advantage for each issue, we conducted a preregistered online survey. Participants were recruited from two culturally distinct societies (Greece and the United States) to test further the assumption that measures of argument advantage are largely independent of the society in which they are obtained.

## 2. Material and methods

The study involved several steps: selection of societies to be included in the study; selection of moral items from the WVS/EVS and ISSP to be used in the study; preregistration of the study of argument advantages; data collection; estimation of global change rates and change rates among richer and poorer societies for each item; estimation of argument advantages; estimation of societies’ moral liberalness; and statistical tests. All data procession, statistical analysis, and data visualization were conducted in R version 4.2.3 [29], including the ‘tidyverse’ [30] collection of packages, and packages ‘DescTools’ [31], ‘boot’ [32], ‘lme4’ [33], ‘ggreppel’ [34] and ‘cowplot’ [35].

### 2.1. Selection of societies

We include all societies participating in the WVS/EVS or ISSP. In the ISSP data for Israel, we exclude the Arab participants because those were not included in the sample at the beginning of the time series. For the same reason, we only used data from the Flemish region in Belgium.

### 2.2. Selection of moral opinion items

**Figure 3.**
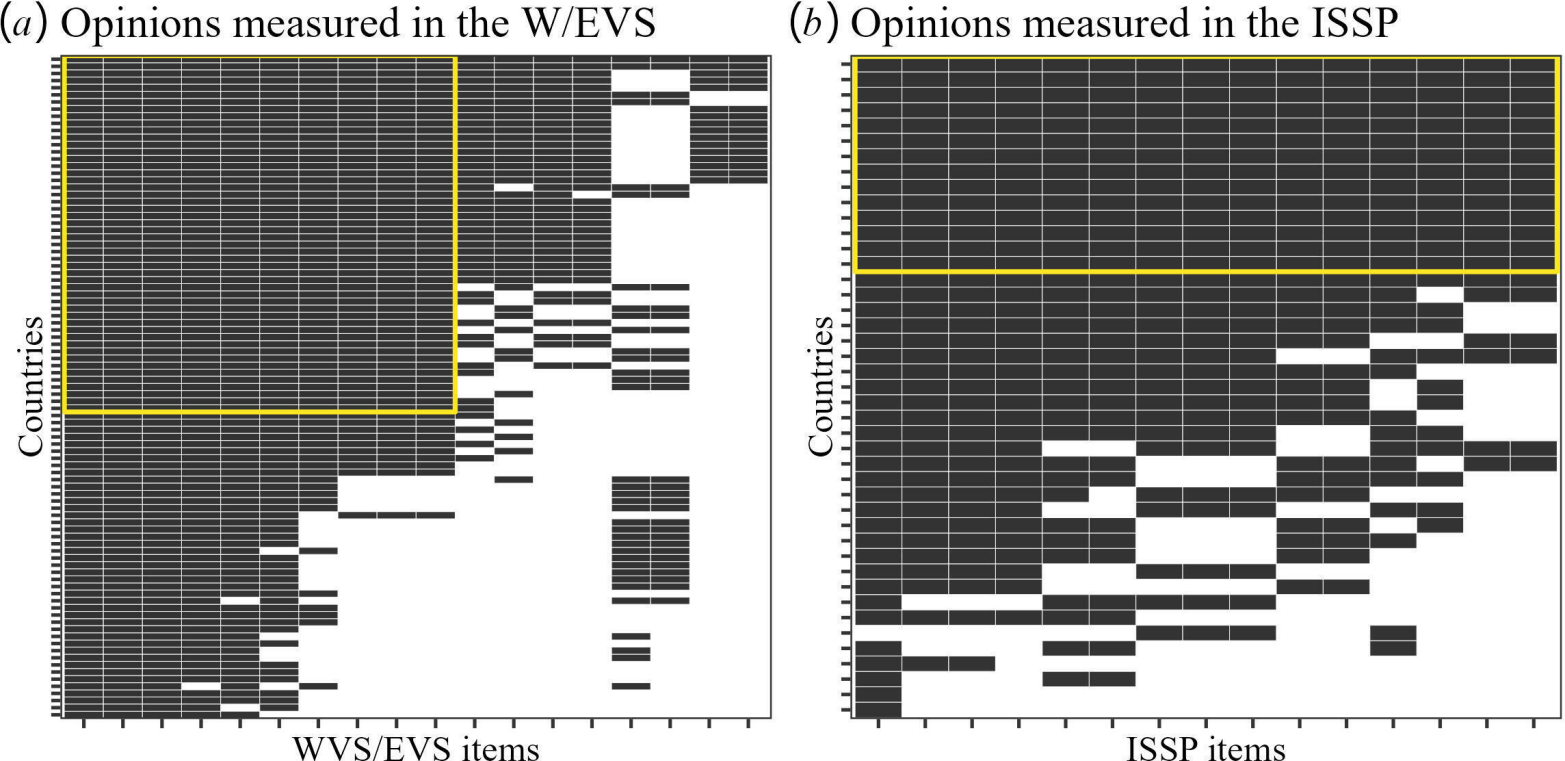
Availability of longitudinal data per society and item. A black cell means that longitudinal data is available in that society for that item in (*a*) WVS/EVS, (*b*) ISSP. The framed rectangles indicate the preregistered selection of combinations of societies and items for the study of argument advantages.

Among the many items in the WVS/EVS and ISSP surveys, we are interested in those included in at least two waves and dealing with moral opinions about right and wrong. We found 18 items in the WVS/EVS and 15 in the ISSP satisfying these criteria (see table 2). For these items, the availability of longitudinal data in each society is shown in figure 3. Sample sizes and the number of times each item has been included in each society are given in electronic supplementary material, tables S1–S4.

**Table 2.** Wordings of moral items in the WVS/EVS and ISSP. Note. The wording of items has been slightly adjusted for the surveys measuring argument advantage, see the WVS and ISSP codebooks to compare with the wording used in the global surveys.

WVS/EVS items
*Divorce*: Do you think that divorce can be justified? *Homosexuality*: Do you think that homosexuality can be justified? *Woman as a single parent:* Do you approve of a woman who wants to have a child as a single parent but does not want to have a stable relationship with a man? *Prostitution*: Do you think that prostitution can be justified? *Abortion*: Do you think that abortion can be justified? *Euthanasia*: Do you think that euthanasia (terminating the life of the incurably sick) can be justified? *Suicide*: Do you think that suicide can be justified? *Parents responsibilities*: Which of the following statements best describes your views about parents’ responsibilities to their children? (A) Parents’ duty is to do their best for their children even at the expense of their own well-being. (B) Parents have a life of their own and should not be asked to sacrifice their own well-being for the sake of their children. *Respect & love for parents*: With which of these two statements do you tend to agree? (A) Regardless of what the qualities and faults of one’s parents are, one must always love and respect them. (B) One does not have the duty to respect and love parents who have not earned it by their behaviour and attitudes. *Following instructions*: People have different ideas about following instructions at work. Some say that one should follow instructions of one’s superiors even when one does not fully agree with them. Others say that one should follow one’s superiors’ instructions only when one is convinced that they are right. Do you think that one should follow instructions of one’s superiors even when one does not fully agree with them? *Sex before marriage:* Do you think that sex before marriage can be justified? *Lying*: Do you think that lying can be justified? *Adultery*: Do you think that adultery can be justified? *Killing in self-defence*: Do you think that it can be justified for someone to kill someone else in self-defence? *Keeping money than one found*: Do you think that it can be justified for someone to keep money that he/she has found? *Parents beating children*: Do you think that it can be justified for parents to beat their children? *Taking soft drugs*: Do you think that taking soft drugs can be justified?

ISSP items
*Protest meetings*: There are many ways people or organizations can protest against a government action they strongly oppose. Do you think organizing public meetings to protest against the government should be allowed? *Protest marches*: There are many ways people or organizations can protest against a government action they strongly oppose. Do you think people should be allowed to organize protest marches and demonstrations against the government? *Cohabit unmarried*: It is alright for a couple to live together without intending to get married. *Convict innocent*: All systems of justice make mistakes, but which do you think is worse: to convict an innocent person or to let a guilty person go free? Do you think that it is worse to convict an innocent person? *Abort when defect wrong*: Do you personally think it is wrong for a woman to have an abortion if there is a strong chance of a serious defect in the baby? *Abort when poor wrong:* Do you personally think it is wrong for a woman to have an abortion if the family has a very low income and cannot afford any more children? *Extramarital sex wrong*: Is it wrong for a married person to have sexual relations with someone other than his or her husband or wife? *Immigrant rights*: There are different opinions about immigrants from other countries living in [respondent’s country]. Do you agree that legal immigrants who are not citizens should have the same rights as citizens? *Racist meetings*: Should people prejudiced against any racial or ethnic group be allowed to hold public meetings? *Marry to have children*: People who want children ought to get married. *Homosex wrong*: Is sexual relations between two adults of the same sex wrong? *Premarital sex wrong*: Do you think it is wrong if a man and a woman have sexual relations before marriage? *Self first*: People should take care of themselves and their families first, before helping other people. *Help poor friends*: People who are better off should help friends who are less well off. *Trad. gender roles*: A husband’s job is to earn money and a wife’s job is to look after the home and family.

### 2.3. Preregistration

The study of argument advantages was preregistered at AsPredicted (https://aspredicted.org/YCY_733). The preregistration specified the subsets of societies and items marked by rectangles in figure 3 (50 societies and 10 items in the WVS/EVS, 15 items and 14 societies in the ISSP), selected so that every society has longitudinal data on every item but excluding societies that strongly restrict freedom of speech and information (specifically, excluding societies whose average World Press Freedom scores over the past 10 years, 2013−2022, were below 55). By mistake, an additional four societies were excluded too (Bosnia, Puerto Rico, Taiwan and Hong Kong). However, it is arguably better to use all available data. Therefore, in addition to the preregistered analysis, we performed analyses that included all societies and items for which data are available (i.e. including all black cells in figure 3).

### 2.4. Data collection

We collected data on argument advantage for all items (i.e. not only the preregistered subset of items). Participants from Greece and the United States were recruited through Prolific to complete a survey in English. As a test of English proficiency and attention, the survey included a 10-item vocabulary test with items selected from the 20-item Gallup-Thorndyke verbal intelligence form A. After excluding 24 participants with less than six correct answers, the final sample consisted of 965 participants, with a mean age of 37.7 years (s.d. = 12.9) and 40.1% women. Split by society, there were 628 participants from the United States (mean age 41.0, s.d. = 13.3, 40.6% women) and 337 participants from Greece (mean age 31.6, s.d. = 9.5, 39.2% women). Each participant was presented with a random sample of the items selected from the WVS/EVS and ISSP. Each item was rated at least 200 times and on average 215 times using a similar procedure as in previous studies [3,11]: participants first stated their position on the item with a dichotomous response scale (e.g. ‘Is it wrong for a married person to have sexual relations with someone other than his or her husband or wife?’ with response scale yes/no). They were then presented with a list of arguments and asked, first, which arguments could be used to support their position and, second, which arguments could be used to support the opposite position. The list of arguments consisted of one argument of each of seven kinds (Harm, Harm-violence, Fairness, Liberty, Authority, Loyalty, Purity), which were drawn from the list in table 1. There was also the option ‘some other reason’. The applicability of each argument to each position was rated on a five-point scale ranging from ‘not at all applicable’ to ‘very applicable’ and coded equidistantly from 0 to 1.

### 2.5. Estimation of moral norm change rates using WVS/EVS and ISSP data

Following prior work [3], we dichotomized all items by combining graded responses and excluding scale midpoints if ambiguous (e.g. for justifiability items in the WVS/EVS, where a 10-step scale was used, we code 7−10 as 1, 1−4 as 0 and exclude 5−6). The dichotomized opinion data consist of 1s and 0s representing agreement and disagreement with the item, respectively. We use all available data from the WVS/EVS and the ISSP for the selected societies. Sample sizes and the number of times an item has been included vary across both items and societies (electronic supplementary material, tables S6–S9). Global opinion change rates were estimated using multi-level logistic regression of opinions on time (in decades) with random intercepts by item and society-item and random slope by item,


(2.1) $$logit(Pr(\mu_{icj}=1))=(\gamma_{000}+u_{0j}+v_{0cj})+(\gamma_{10}+u_{1j})tim_{ecj},$$

where μ*_icj_* is the dichotomized opinion data for individual *i* in society *c* on item *j*. The sum (*γ*_10_*+u*_1*j*_) is the estimated global change rate for item *j*. All models apply the survey weights provided.

To estimate change rates in different time periods, we used society-items that were measured at least three times. We ran the model (2.1) twice, once with data from the first time point to the time point closest to the middle, and once with data from the time point closest to the middle to the last time point.

For the preregistered analysis, which uses only items and societies so that every society has longitudinal data on every item, we estimate global opinion change using logistic regression with time (measured in 10 years units) as the main predictor and adjusted for society differences with a series of dummy variables.

### 2.6. Estimation of argument advantages using our survey data

A participant’s argument advantage score for a given opinion was calculated as their average applicability rating of the Harm, Fairness and Liberty arguments of that opinion minus their average applicability rating of the Harm, Fairness and Liberty arguments of the opposing opinion. (For the preregistered analysis, we also included the Violence arguments in the same average.) These scores were then aggregated across all participants in a sample.

### 2.7. Estimation of binding argument advantages using our survey data

For illustrative purposes, we similarly calculated the ‘binding argument advantage’ of a given opinion as the average applicability rating of the Authority, Loyalty and Purity arguments of that opinion minus the average applicability rating of the Authority, Loyalty and Purity arguments of the opposing opinion.

### 2.8. Estimation of the moral liberalness of societies using data from cross-cultural Moral Foundations Questionnaire studies

The MFQ measures people’s reliance on different moral foundations [7]. The MFQ consists of two sets of items: judgements (e.g. ‘chastity is an important and valuable virtue’) and relevance items asking participants how relevant various concerns are for their moral judgements (e.g. ‘whether or not someone violated standards of purity and decency’). To estimate societies’ moral liberalness, we use data from three cross-cultural studies of moral foundations. Klein *et al*. used only the 15 relevance items (with responses on a 6-point scale) in a study with 6966 participants in 30 countries [26]. Pagliaro *et al*. used the full 30-item version of the MFQ (with responses on 7-point scales) in a study with 6644 participants in 22 countries [27]. Saucier *et al*. used a shorter 20-item version (with responses on 5-point scales) in a study with 8883 participants in 33 countries [28]. Each item is associated with one moral foundation. To estimate a society’s moral liberalness, we standardize all response scales to have a range between 0 and 1, aggregate responses within each country and each study, and calculate the average response to all items for individualizing foundations (harm and fairness) minus the average response to all items for binding foundations (authority, in-group loyalty and purity). For any country included in more than one study, we use the average measure from those studies. In this way, measures of moral liberalness are obtained for 48 societies used in this study.

### 2.9. Statistical tests

In all statistical tests on profiles, we include both opinions on each issue, meaning the profile will consist of pairs of values with opposite signs. When calculating bootstrap confidence intervals, the resampling units are issues (pairs of opinions).

We used the concordance correlation coefficient (CCC) to analyse the agreement about the profiles of argument advantages between different societies (United States and Greece), different genders (male and female), and different age groups (younger versus older than the median age).

Each society only has data on a subset of the issues. To test prediction 1 (that different societies have similar profiles of norm change for moral issues), we partition the countries randomly into two groups of equal size, calculate the aggregated profile of change within each group, and compute the correlation between these two group-level profiles. We repeat this 300 times. We test that, regardless of how societies are grouped, we always find a strong (Pearson) correlation between the group-level profiles of change.

To test prediction 2 (that the profile of norm change is consistent across different time periods), we focus on 25 items that were measured at least three times so that change can be estimated separately in an earlier and a later period. We test that the global mean change profiles obtained in each period are positively correlated, using the Pearson correlation.

Prediction 3 (that argument advantage measures account for the global profile of norm change) was preregistered. The preregistered analysis focused on two smaller sets of issues and societies, one for the WVS/EVS and one for the ISSP, selected so that longitudinal data was available for every item in every society. For each set, we test that the global mean change profile is correlated with the argument advantage values, using the Pearson correlation. As a complement to the preregistered analysis, we test that the global mean change profile obtained using all available data is correlated with the argument advantage values. We also perform the same analysis using the *binding* argument advantage instead to examine whether it is specifically an advantage with respect to individualizing arguments that is correlated with norm change in that direction.

Predictions 4 and 5 concern how the moral liberalness of societies moderates the effects of argument advantage on norms at the first point they are measured and norm change from that point onward. We test both predictions in the same extension of the multi-level logistic model (1.1)_,_


$$logit(Pr(\mu_{(ic)j}=1))=(\gamma_{00}+u_{0j}+v_{0cj})+(\gamma_{10}+u_{1j})time_{cj}+(\gamma_{20}+u_{2j})LIB_{c}+(\gamma_{30}+u_{3j})time_{cj}\times LIB_{c}+\gamma_{01{AA}_{j}}+\gamma_{11}time_{cj}\times AA_{j}+\gamma_{21}time_{cj}\times AA_{j}+\gamma_{31}LIB_{c}\times AA_{j}+\gamma_{41}time_{cj}\times LIBc\times AA_{j},$$

where LIB_c_ is the measure of the moral liberalness of society c. The variable time_cj_ is calibrated so that it has value 0 in the first year issue j was measured in any country. We test that the coefficient of LIB_c_ × AA_j_ is positive (prediction 4) that the coefficient of time_cj_ × LIB_c_× A_Aj_ is positive (prediction 5).

## 3. Results

### 3.1. The moral liberalness of societies

**Figure 4.**
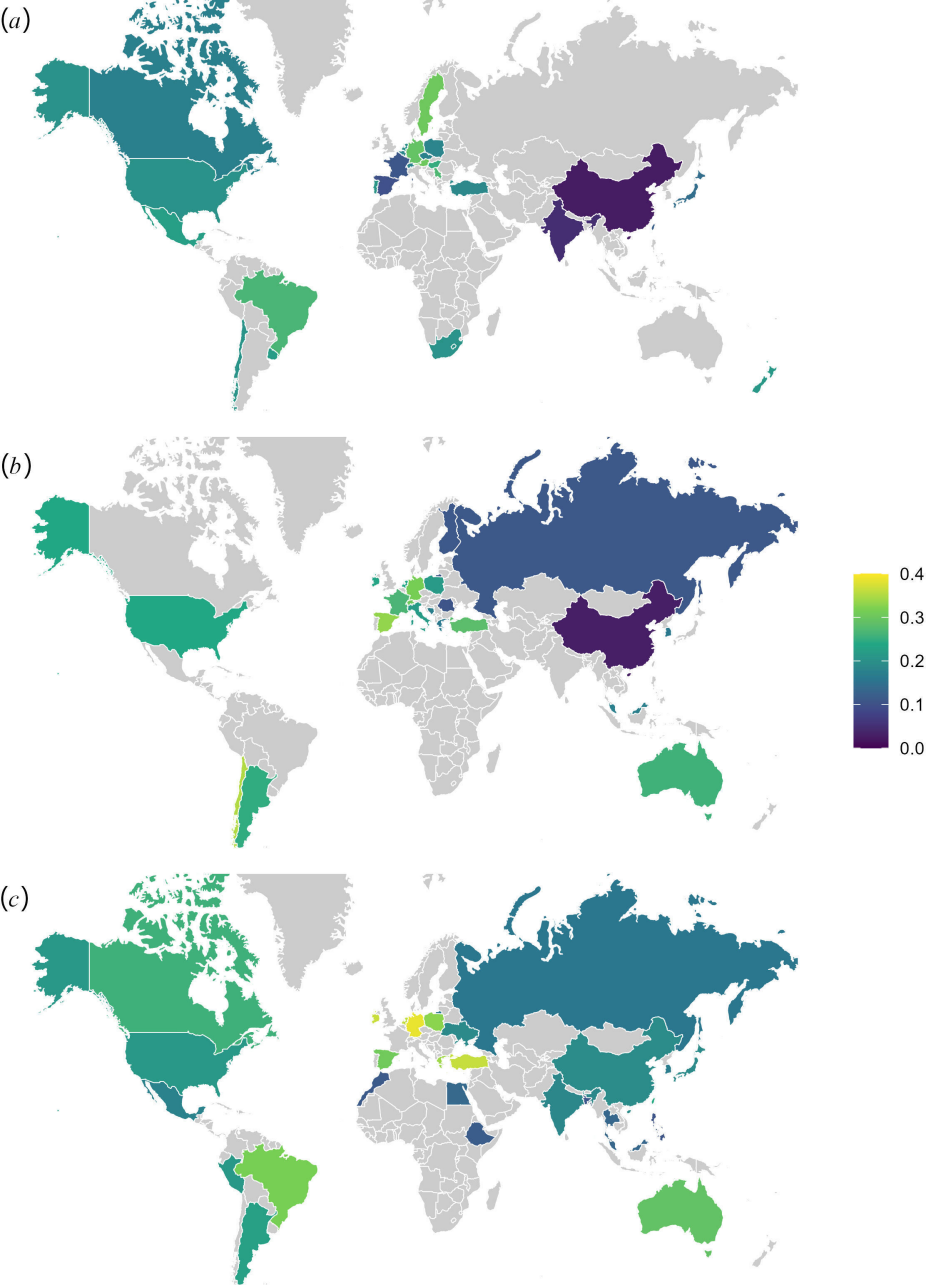
The average difference in reliance scores between individualizing and binding foundations in (*a*) 27 societies surveyed by Klein *et al*. [26], (*b*) 25 societies surveyed by Pagliaro *et al*. [27], (*c*) 30 societies surveyed by Saucier *et al*. [28].

For societies around the world, figure 4 shows the average difference in reliance scores between individualizing and binding foundations obtained from three data sources. As mentioned in the introduction, all differences are positive but vary considerably in size across societies.

### 3.2. The argument advantage of opinions on various moral issues

For each of the 33 moral issues in our study, one opinion was arbitrarily selected to be positively coded. Table 3 reports the argument advantage of the positively coded opinions, estimated separately in Greece and the United States. A negative argument advantage means that the opposite opinion holds the argument advantage. In support of our assumption that argument advantages are largely society-independent, the similarity between estimates obtained in the two societies was close to perfect, CCC = 0.96, 95% CI [0.93, 0.98], across *n* = 32 moral issues. Argument advantage measures were also similar when estimated separately among men and women, CCC = 0.99, 95% CI [0.97, 0.99], or estimated separately among younger and older participants, CCC = 0.97, 95% CI [0.95, 0.98].

**Table 3.** Estimates of argument advantages and norm change rates for 33 behaviours. Note. Change refers to change rates in log odds per 10 years. They were estimated in two ways: ‘prereg.’ refers to the preregistered analysis of 25 items using a subset of data, and ‘alter.’ refers to the alternative analysis using all available data.

item	data source	no. countries	arg. adv.	M prereg. change	CI prereg. change	M alter. change	CI alter. change
*homosexuality*	WVS/EVS	92	0.57	0.66	0.59, 0.72	0.64	0.64, 0.65
*divorce*	WVS/EVS	93	0.44	0.39	0.34, 0.45	0.36	0.35, 0.37
*woman single parent*	WVS/EVS	70	0.43	0.23	0.17, 0.30	0.21	0.20, 0.22
*sex before marriage*	WVS/EVS	31	0.35			0.22	0.17, 0.27
*prostitution*	WVS/EVS	87	0.33	0.23	0.17, 0.29	0.25	0.24, 0.26
*enjoying sexual freedom*	WVS/EVS	42	0.33			0.25	0.22, 0.28
*taking soft drugs*	WVS/EVS	45	0.23			0.53	0.51, 0.55
*abortion*	WVS/EVS	93	0.20	0.28	0.22, 0.34	0.24	0.23, 0.25
*euthanasia*	WVS/EVS	91	0.19	0.30	0.25, 0.36	0.23	0.23, 0.24
*suicide*	WVS/EVS	93	0.18	0.22	0.16, 0.29	0.23	0.22, 0.24
*adultery*	WVS/EVS	37	0.07			−0.25	−0.28, −0.23
*lying*	WVS/EVS	38	0.01			−0.07	−0.10, −0.05
*keeping money that one found*	WVS/EVS	16	0.00			0.34	0.27, 0.41
*parents’ duty even at own expense*	WVS/EVS	60	−0.08	0.18	0.11, 0.24	0.16	0.15, 0.17
*killing in self-defence*	WVS/EVS	16	−0.14			0.00	−0.05, 0.05
*respect & love for parents*	WVS/EVS	60	−0.15	−0.08	−0.14, −0.02	−0.07	−0.08, −0.06
*parents beating children*	WVS/EVS	35	−0.28			0.14	0.08, 0.21
*following instructions at work*	WVS/EVS	60	−0.32	−0.03	−0.09, 0.03	0.00	−0.01, 0.01
*immigrant having same rights*	ISSP	25	0.47	−0.16	−0.37, 0.05	−0.24	−0.27, −0.21
*cohabiting unmarried*	ISSP	34	0.45	0.18	0.08, 0.29	0.14	0.13, 0.16
*organizing protest marches*	ISSP	31	0.39	0.17	0.09, 0.25	0.14	0.12, 0.16
*organizing protest meetings*	ISSP	31	0.38	0.08	0.02, 0.14	0.07	0.05, 0.09
*convicting innocent worse*	ISSP	31	0.31	−0.02	−0.10, 0.06	−0.06	−0.08, −0.05
*helping friends who are less well off*	ISSP	20	0.08	−0.02	−0.09, 0.06	−0.07	−0.09, −0.05
*holding meetings against an ethnic group*	ISSP	29	−0.06	0.08	−0.10, 0.27	0.17	0.13, 0.21
*extramarital sex wrong*	ISSP	37	−0.13	0.05	−0.09, 0.19	0.01	0.03, 0.08
*abortion because of defect wrong*	ISSP	30	−0.14	0.01	−0.08, 0.09	0.06	−0.00, 0.03
*taking care of self before helping others*	ISSP	20	−0.15	0.07	−0.01, 0.15	0.08	0.05, 0.10
*abortion because of low income wrong*	ISSP	36	−0.19	−0.04	−0.15, 0.07	−0.04	−0.05, −0.03
*marry to have children*	ISSP	35	−0.31	−0.37	−0.48, −0.26	−0.37	−0.40, −0.37
*premarital sex wrong*	ISSP	30	−0.35	−0.13	−0.25, −0.02	−0.20	−0.22, −0.17
*follow traditional gender roles*	ISSP	42	−0.45	−0.34	−0.41, −0.27	−0.38	−0.40, −0.37
*homosexual relations wrong*	ISSP	37	−0.53	−0.49	−0.58, −0.41	−0.50	−0.51, −0.49

### 3.3. Change rates of norms on various moral issues

For each of the 33 items, one opinion was arbitrarily selected to be coded positive and the change rate of the popularity of this opinion was estimated in each society. Change rates are measured in log odds per 10 years, where log refers to the natural logarithm and odds refer to the proportion of people who hold the opinion divided by the proportion of people who do not. A positive (versus negative) change rate represents an increase (versus decrease) in popular support for the positively coded opinion. For example, the opinion that homosexuality is justifiable has a global mean change rate of 0.65, which means that over 10 years the ratio between the proportion of people who judge homosexuality as justifiable and the proportion of people who do not has changed by a factor exp(0.65) = 1.9. Table 3 reports the global change rates obtained using two different analyses: the preregistered analysis, which uses subsets of the data, and the alternative analysis that uses all available data.

### 3.4. Tests of predictions made by moral argument theory

In line with prediction 1, the correlation between the mean change profiles for different groups of societies was consistently strongly positive, ranging between *r* = 0.57 and *r* = 0.95, across 300 random partitions of the societies into two equally sized groups.

In line with prediction 2, there was a strong positive correlation between the global profiles of mean changes in the earlier period and the later period, *r* = 0.66, 95% CI [0.38, 0.86], across *n* = 25 issues.

In line with prediction 3, the preregistered analysis yielded a positive correlation between argument advantage and global mean change rates in the WVS/EVS data*, r* = 0.87, 95% CI [0.53, 0.97], across *n* = 10 issues, as well as in the ISSP data, *r* = 0.54, 95% CI [0.04, 0.83], across *n* = 15 issues. This finding was replicated when we performed an analysis using all available data, *r* = 0.63, 95% CI [0.36, 0.83], across *n* = 33 items. Thus, the finding that global norm change is predicted by measures of argument advantage was robust across different datasets and analytical choices. Moreover, consistent with the theory that moral norm change is driven specifically by individualizing arguments, the correlation between global norm change and *binding* argument advantage is negative, *r* = −0.65, 95% CI [−0.81, −0.46].

Table 4 presents the results from a mixed-level (societies and issues) logistic model of norms with a three-way interaction of time, the argument advantage (AA) of 33 issues, and the moral liberalness (LIB) of 48 societies. In line with prediction 4, the effect of argument advantage on norms at the first time of measurement is stronger in societies that are more morally liberal (i.e. the coefficient for AA × LIB is positive). In line with prediction 5, the effect of argument advantage on norm change over time is stronger in societies that are more morally liberal (i.e. the coefficient for time × AA × LIB is positive).

**Table 4.** Results from mixed-level logistic models of norms with a three-way interaction of time, argument advantage (AA) and moral liberalness (LIB).

	estimate	*p*
fixed effects		
intercept	−0.56 [−1.08, −0.04]	0.036
time	0.04 [−0.03, 0.11]	0.271
AA (issue level)	−1.24 [−2.94, 0.46]	0.152
LIB (society level)	−0.02 [−0.13, 0.09]	0.767
AA × LIB	1.10 [0.74, 1.45]	<0.001
time × AA	0.48 [0.26, 0.71]	<0.001
time × LIB	0.00 [−0.05, 0.04]	0.842
time × AA × LIB	0.17 [0.02, 0.32]	0.029
random effects		
s.d. society : issue	0.77	
s.d. issue	1.49	
s.d. time by issue	0.20	
s.d. LIB by issue	0.25	
s.d. time × LIB by issue	0.13	
num. obs.	3664	
N issue	33	
N society : issue	965	
R2 marg.	0.071	

**Figure 5.**
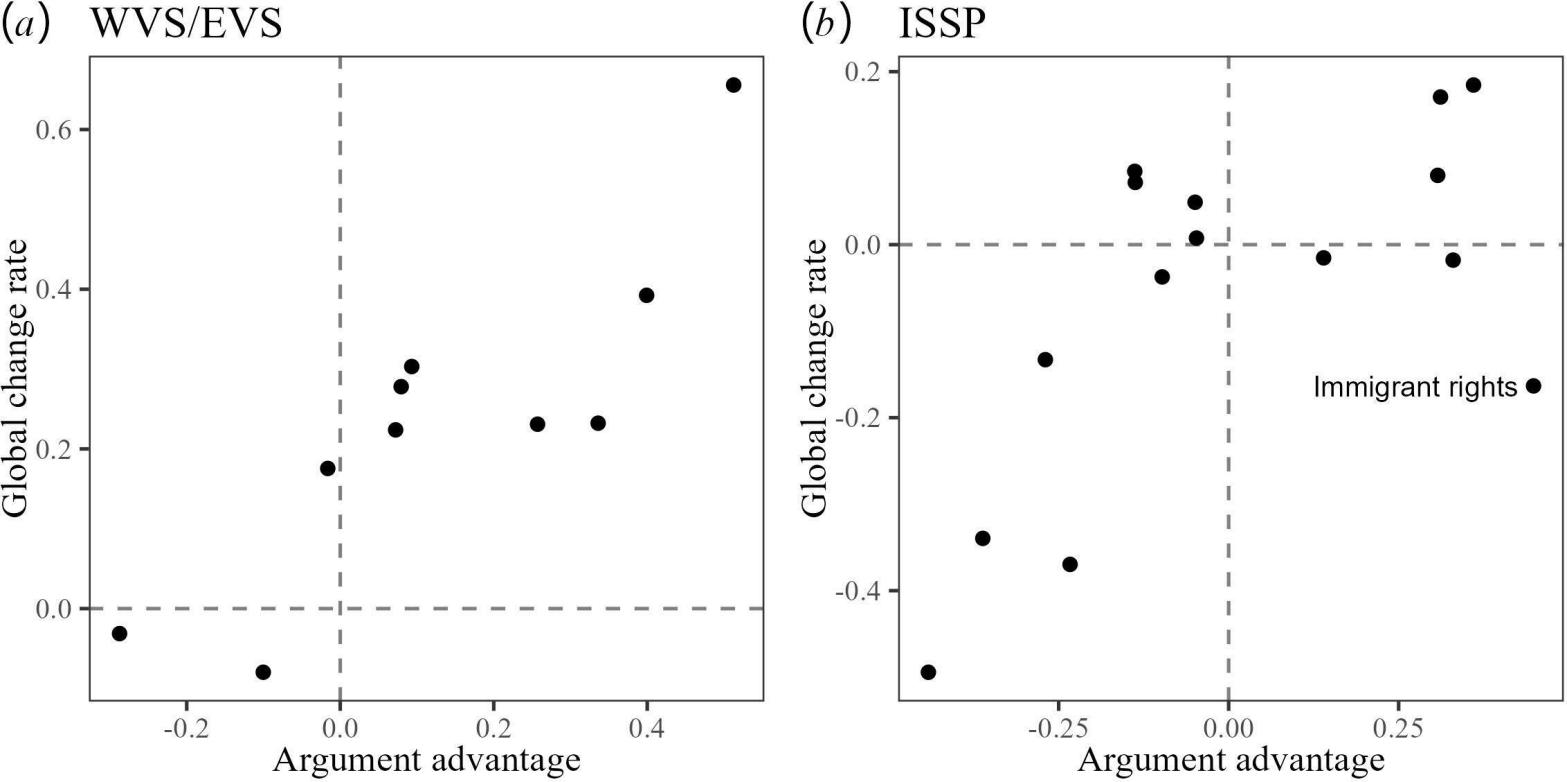
Scatter plots illustrating the key finding of a strong correlation between the global profile of moral norm change and the argument advantage values for the same norms. The global profile of moral norm change is estimated in two datasets: (*a*) WVS/EVS data on 10 issues in 50 societies, (*b*) ISSP data on 15 issues data in 14 societies. Each dot represents a moral issue. Dashed reference lines at zero are included to highlight that the norm change and the argument advantage have the same sign for most issues. An outlier is labelled.

Note that the mixed-level analysis additionally yielded a positive coefficient for time × AA. This is a replication of the earlier result (prediction 3) that norm change is correlated with argument advantage. In these analyses, the issues are the units of analysis. To see whether some issues are outliers, we plotted the norm change rates (obtained in the preregistered analysis) against the argument advantage for WVS/EVS issues (figure 5*a*) and ISSP issues (figure 5*b*). The figure highlights an outlier: the issue of immigrant rights, for which the argument advantage yields a clearly incorrect prediction about the global norm change. We return to this prediction miss in the discussion.

## 4. Discussion

Any number of factors could influence moral norms around the world. Is it nonetheless possible to predict, with any accuracy, how norms change globally for a variety of moral issues? In this study, we derived such predictions from the moral argument theory. This theory describes a within-generation cultural evolutionary process based on people exchanging arguments on moral issues. In this process, moral norms change gradually but consistently in the direction that has the ‘argument advantage’, that is, the direction that is best justified by individualizing arguments. This gradual change happens because individualizing arguments tend to be relevant to everyone whereas arguments based on the binding moral foundations only tend to be relevant to moral conservatives and not to moral liberals. The more moral liberals there are in a society, the faster its moral norms will change in this process.

Predictions from the theory proved quite accurate when tested in available cross-cultural longitudinal data on norms on 33 moral issues in 94 societies. First, we found similar norm change profiles in different societies. Thus, it is meaningful to talk about a global profile of moral norm change. This global profile includes, for example, a decreasing acceptance of adultery and lying; a decreasing importance of traditional gender roles and marriage; and an increasing acceptance of homosexual relations, taking soft drugs, keeping money that one found and organizing protests. Second, the global change profile was similar when estimated in two different periods. Thus, there is a consistent direction of change over time. Third, measures of the argument advantage of each issue account for the global profile of moral norm change. In other words, global norms have indeed been moving in the direction best justified by individualizing arguments. Fourth, this move has gone faster in more morally liberal societies, as predicted by moral argument theory. Fifth, norms in more morally liberal societies had moved further in this direction already at the first time of measurement.

It is possible that the predictions could be further improved. The measures of argument advantages we used were only obtained in the United States and Greece and only at the current time. Both countries provided similar measures, which is consistent with the assumption that the same measures of argument advantages may apply across societies and time. However, this assumption can only be approximately true. If we had perfect measures of argument advantages in every society at every point in time, they could be input into the computational model to yield predictions that should be even better.

While measures of argument advantage generally predicted how global norms have changed, the issue of whether legal immigrants should be given the same rights as citizens stood out as a clear prediction miss. Namely, while our survey found that individualizing arguments favour giving rights to immigrants, ISSP data show that public support for doing so has decreased over time. A possible reason for this discrepancy is that the moral argument theory does not consider changing realities, and this may be especially pertinent to the immigrant issue as the number of migrants has increased globally [36]. With the increasing number of immigrants, people may have increasing concerns about the socioeconomic consequences of immigration [37], which are not captured by moral arguments. At any rate, the interesting result is not that the theory makes a miss but that it mostly makes correct predictions about global norm change. As an extremely complex set of factors potentially influences global norm change, it might not have been predictable at all.

As far as we know, no other theory has made specific predictions about how global norms change for any moral issue, although they possibly could. For example, consider world society theory [38,39]. World society theory is a top-down theory positing that certain norms, values and beliefs among a global elite form a normative framework that exerts considerable influence on actors in the international arena. To gain legitimacy and acceptance on the global stage, states are assumed to adjust their behaviour to align with these elite norms. This may in turn influence norms in the local populations. Thus, world society theory provides a mechanism for global norm change. It would be interesting if future work in world society theory would state which predictions it makes about the change of each moral norm covered in the present study. Another theory of global norm change is modernization theory; specifically, the ‘post-materialist thesis’ of Inglehart [40–42]. In brief, the thesis is that as societies modernize economically and become more prosperous, a shift in values occurs such that materialist concerns (e.g. economic stability and security) are increasingly replaced by post-materialist values (e.g. self-expression, individual rights and environmental sustainability). As societies undergo similar stages of economic development, this fosters a global trend of increasingly post-materialist values, which can be conceived as moral liberalism (i.e. an emphasis on individualizing foundations over binding foundations). This thesis is consistent with life history studies finding that growing up in stability and security correlates with a greater reliance on individualizing foundations [43,44]. Some researchers in modernization theory have provided lists of norms that are expected to change in a certain direction. However, as far as we know, there has been no attempt to explain the differences in change rates across issues. In sum, we are still waiting for other theoretical approaches to yield testable predictions about global change in multiple specific norms.

Moral argument theory makes testable predictions of differences at many levels: between issues, between societies and between different groups within a society. The accumulated evidence from such tests shows both that the theory makes accurate predictions and that it is useful to understand a broad set of phenomena, including how norms change on hundreds of moral issues [3,11], prediction of future change [25], how moral norms differ between ideological groups [13], across different levels of cognitive ability [24] and across different levels of political discussion frequency [12], in addition to the current study of how moral norms and norm change rates differ between societies.

In this study, we have used all longitudinal data available on moral norms from the two best cross-cultural sources of survey data, we could find (the International Social Survey Program and the World Values Survey/European Values Study). Nonetheless, the Global South is less well represented than the Global North. Future waves of these studies will hopefully address this limitation.

In conclusion, our findings reveal a global pattern of time trends in moral norms that is well predicted by the moral argument theory. This study contributes to several areas of behavioural science, including moral psychology, cultural psychology and cultural evolution. It also speaks to the emerging field of historical psychology, which emphasizes that the roots of cross-cultural variation often lie in the past [45]. Consistent with that view, our study showed that cross-cultural variation in moral norms can be understood as the outcome of societal differences in the rates at which norms have changed up to that time. The theory further predicts that norms will continue to change in the argument-advantaged direction, thereby contributing to research about the future as well. In all, this study shows that an empirically validated theory of global norm change is achievable.

## Data Availability

The data from the WVS/EVS [46,47] and ISSP [48–52] are publicly available at their respective websites (https://www.worldvaluessurvey.org/wvs.jsp and https://issp.org/). Data created in this study, as well as code and materials, are available at [53].
